# Crosstalk between Androgen-ZIP9 Signaling and Notch Pathway in Rodent Sertoli Cells

**DOI:** 10.3390/ijms21218275

**Published:** 2020-11-05

**Authors:** Alicja Kamińska, Sylwia Marek, Laura Pardyak, Małgorzata Brzoskwinia, Barbara Bilinska, Anna Hejmej

**Affiliations:** 1 Department of Endocrinology, Institute of Zoology and Biomedical Research, Faculty of Biology, Jagiellonian University, 30-387 Kraków, Poland; ala.kaminska@uj.edu.pl (A.K.); s.marek@doctoral.uj.edu.pl (S.M.); laura.pardyak@urk.edu.pl (L.P.); m.brzoskwinia@doctoral.uj.edu.pl (M.B.); barbara.bilinska@uj.edu.pl (B.B.); 2 Center of Experimental and Innovative Medicine, University of Agriculture in Krakow, 30-248 Kraków, Poland

**Keywords:** Notch, androgen, Sertoli cell, testis

## Abstract

Our recent study demonstrated altered expression of Notch ligands, receptors, and effector genes in testes of pubertal rats following reduced androgen production or signaling. Herein we aimed to explore the role of nuclear androgen receptor (AR) and membrane androgen receptor (Zrt- and Irt-like protein 9; ZIP9) in the regulation of Notch pathway activation in rodent Sertoli cells. Experiments were performed using TM4 and 15P-1 Sertoli cell lines and rat primary Sertoli cells (PSC). We found that testosterone (10^−8^ M–10^−6^ M) increased the expression of *Notch1* receptor, its active form Notch1 intracellular domain (N1ICD) (*p* < 0.05, *p* < 0.01, *p* < 0.001), and the effector genes *Hey1* (*p* < 0.05, *p* < 0.01, *p* < 0.001) and *Hes1* (*p* < 0.05, *p* < 0.001) in Sertoli cells. Knockdown of AR or ZIP9 as well as antiandrogen exposure experiments revealed that (i) action of androgens via both AR and ZIP9 controls *Notch1*/N1ICD expression and transcriptional activity of recombination signal binding protein (RBP-J), (ii) AR-dependent signaling regulates *Hey1* expression, (iii) ZIP9-dependent pathway regulates *Hes1* expression. Our findings indicate a crosstalk between androgen and Notch signaling in Sertoli cells and point to cooperation of classical and non-classical androgen signaling pathways in controlling Sertoli cell function.

## 1. Introduction

Mammalian testis sperm production is a process supported and controlled by somatic cells of seminiferous epithelium, Sertoli cells. These cells are considered to be key mediators of androgen action in the control of spermatogenesis. In seminiferous tubules, the expression of androgen receptor (AR), a member of nuclear receptor subfamily 3, is restricted to Sertoli cells [1,2] and selective ablation of the AR in these cells leads to spermatogenic arrest, indicating that AR in Sertoli cells is crucial to maintain spermatogenesis [3]. Binding of testosterone (major testicular androgen produced by interstitial tissue of the testis) to the AR triggers classical signaling pathway, inducing nuclear localization, dimerization, and interaction of hormone-receptor complex with androgen response element, a DNA sequence located in the regulatory region of androgen regulated genes [4]. In addition, alternative pathways may account for rapid effects of androgens via cytoplasmic or membrane-localized AR [5]. It is now well established that in adult males AR signaling in Sertoli cells is required for Sertoli cell maturation, the maintenance of the blood-testis barrier, Sertoli cell-spermatid adhesion, completion of meiosis and spermiation [6].

In 2014, a member of the Zrt and Irt-like protein zinc transporter family ZIP9 was demonstrated to display specific, high-affinity binding of testosterone. Both functions of ZIP9, zinc transport and testosterone-induced activation of second messengers, are mediated through G proteins [7]. In AR-deficient rat Sertoli cell line, 93RS2, testosterone binding to ZIP9 stimulated extracellular signal-regulated protein kinase 1/2 (ERK1/2), cAMP-response element binding protein (CREB), and activating transcription factor 1 (ATF-1) phosphorylation and regulated the expression of tight junction proteins claudin-1 and claudin-5 [8]. Recently, we have found that ZIP9 is also localized to Sertoli cells in mouse and bank vole seminiferous epithelium [9,10]. In bank vole, a seasonally breeding rodent, expression level of ZIP9 during reproductive quiescence was correlated with testes regression status [10]. In dogs, the levels of ZIP9 in the testes were abrogated after long-term gonadotropin-releasing hormone-agonist treatment, which indicates that expression of this protein depends on the activity of hypothalamic-pituitary-gonadal axis [11]. The role of ZIP9 in testis pathology has not been described yet. To date, the presence of ZIP9 and its involvement in the regulation of apoptosis [12] and metastasis [13] of prostate and breast cancer cells have been demonstrated. However, it has been recently found that ZIP9 immunoreactivity does not show a significant difference between cancerous and non-cancerous breast and prostate specimens [14].

Apart from hormonal signals, Sertoli cell physiology is controlled by local interactions, including paracrine signaling, gap junctional communication and contact-dependent signaling [15]. The last of these is represented by a Notch signaling pathway triggered by the interaction of Notch receptors with Jagged or Delta-like ligands localized in the plasma membranes of adjacent cells. Proper Notch pathway activity in Sertoli cells is crucial for determination of germ cell fate during early testis development and controls spermatogonial differentiation in adult testis [16,17]. Notch1/HEY1/HES1 pathway is involved in the control of glial-derived neurotrophic factor production and *Cyp26b1* expression in Sertoli cells [17,18]. Disruption of Notch signaling in adult testis results in increased apoptosis and spermatogenesis defects [19].

In contrast to many signaling pathways, Notch pathway lacks intermediate steps and does not exploit downstream secondary messengers for signal amplification. Binding of a ligand to Notch receptor results in receptor cleavage and translocation of Notch intracellular domain (NICD) to the nucleus, where it interacts with a transcription factor recombination signal binding protein (RBP-J), and Mastermind-like 1 [20]. The Notch co-activator complex induces the expression of target genes belonging to hairy/enhancer of split (Hes) and Hes-related with YRPW (Tyr-Arg-Pro-Trp) motif 1 (Hey) families [21].

Despite its simplicity, outcomes of the pathway activation may be diverse even in the same cell type due to its complex regulation at different levels [22,23]. Studies using various tissues and cellular models demonstrated that this pathway may also crosstalk with other signaling pathways and such interactions may determine the final outcome [24,25,26,27].

Androgens were identified as factors modulating activity of Notch signaling in several cell types. Long-term testosterone enanthate treatment increased the expression of activated Notch1 in muscle satellite cells of older men consistent with increased satellite cell replication [28]. The importance for androgen-Notch interaction for the course of prostate development and morphogenesis was also demonstrated [29,30]. DeFalco et al. [31] found that in mice testosterone plays a role in maintaining the balance between progenitor cells and differentiated fetal Leydig cells, regulating levels of JAG1 and Notch2. In respect to androgen action in Sertoli cells, particular attention was devoted to genes related to the development of the blood-testis barrier [32]. Our recent study demonstrated that Notch pathway in Sertoli cells is involved in the regulation of blood-testis barrier proteins, claudins, in androgen-dependent manner. Using RNAi technique to reduce the expression of androgen receptors, we provided evidence that the effects of Notch signaling on claudin-5 and claudin-11 are dependent on the activation of ZIP9 or AR, respectively [9]. Notably, this was the first report on ZIP9 regulation by the Notch pathway. We have also found that reduced androgen production or signaling alters the expression of Notch receptors, ligands and effector genes in testes of pubertal rats [33]. To provide deeper insight in androgen-Notch crosstalk in mammalian testis and reveal the mechanisms involved in this interaction, we aimed to explore the role of nuclear and membrane androgen receptors in the regulation of Notch pathway activation in rodent Sertoli cells in vitro.

## 2. Results

**Figure 1:**
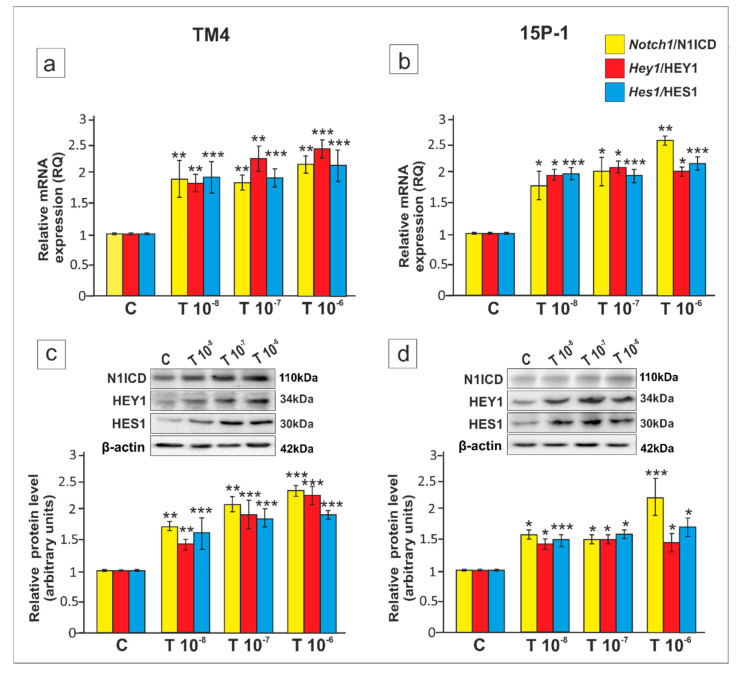
The effect of testosterone on *Notch1*, *Hey1* and *Hes1* mRNA, and Notch1 intracellular domain (N1ICD), HEY1 and HES1 protein expression in TM4 and 15P-1 Sertoli cell lines. Cells were treated with a vehicle (control, C), 10^−8^ M, 10^−7^ M or 10^−6^ M testosterone (T) for 24 h (**a**,**b**) Relative expression of mRNAs (RQ) was determined using quantitative real-time RT-PCR analysis. The expression values of the individual genes were normalized to the mean expression of *Rn18s*, *B2m*, *Gapdh* and *Actb*. (**c**,**d**) Western blot detection of the proteins. The relative level of studied proteins was normalized to β-actin. The protein levels within the control group were arbitrarily set at 1. The histograms are the quantitative representation of data (mean ± SD) of three independent experiments, each in triplicate. Significant differences from control values are denoted as * *p* < 0.05, ** *p* < 0.01, and *** *p* < 0.001.

Firstly, to investigate whether Notch pathway activity in Sertoli cells is directly regulated by androgens, relative level of *Notch1* expression, activated form of Notch1 receptor (N1ICD) and the expression of the target genes *Hey1* and *Hes1* were determined in two murine Sertoli cell lines TM4 (from 11–13 day-old mice; expressing both the AR and ZIP9) and 15P-1 (from 6 month-old mice; lacking the AR, but expressing ZIP9). We found that testosterone, in the range of serum and testicular concentrations 10^−8^ M–10^−6^ M [34,35,36], increased mRNA and protein expression of *Notch1*/N1ICD (*p* < 0.05, *p* < 0.01, *p* < 0.001), *Hey1* (*p* < 0.05, *p* < 0.01, *p* < 0.001) and *Hes1* (*p* < 0.05, *p* < 0.001) in both cell lines (Figure 1a–d). The lowest concentration capable of effectively activating Notch pathway (10^−8^ M) was used in further experiments. 

**Figure 2:**
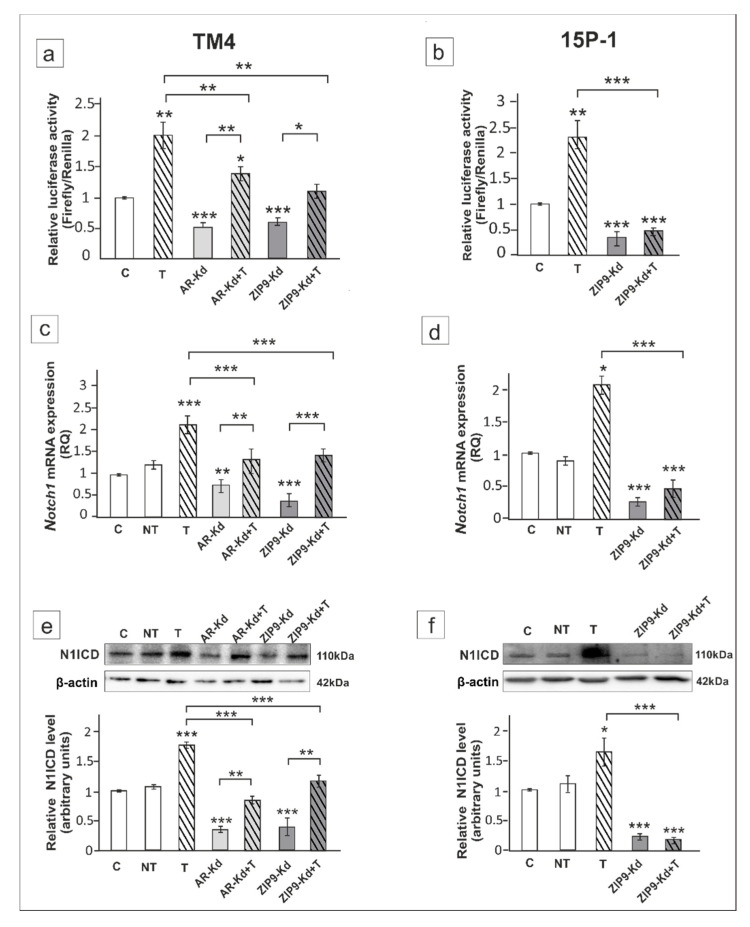
The effect of androgen receptor (AR) and ZIP9 knockdown on Notch pathway activity, *Notch1* mRNA and N1ICD expression in TM4 (**a**,**c**,**e**) and 15P-1 (**b**,**d**,**f**) Sertoli cells. (**a**,**b**) Cells were transfected with 2 × 10^−12^ M of recombination signal binding protein (RBP-J) reporter and 5 × 10^−8^ M non-targeting siRNA (control), AR-siRNA (AR-Kd) or ZIP9-siRNA (ZIP9-Kd). After 16 h cells were treated with 10^−8^ M testosterone (T) or vehicle (C). Cells were harvested after a 24 h incubation and the ratio of firefly-derived luminescence over renilla-derived luminescence was determined. Data are means ± SD for a single experiment done in triplicate. (**c**–**f**) Cells were treated with transfection reagent alone (C), transfection reagent + non-targeting siRNA (negative control, NT), transfection reagent + AR siRNA or ZIP9 siRNA. After 24 h 10^−8^ M T or vehicle was added to the culture. (**c**,**d**) Relative expression of mRNAs (RQ) was determined using real-time RT-PCR analysis. The expression values of the individual genes were normalized to the mean expression of *Rn18s*, *B2m*, *Gapdh* and *Actb*. (**e**,**f**) Western blot detection of the proteins. The relative level of studied protein was normalized to β-actin. The protein levels within the control group were arbitrarily set at 1. The histograms are the quantitative representation of data (mean ± SD) of three independent experiments, each in triplicate. Significant differences from control values are denoted as * *p* < 0.05, ** *p* < 0.01, and *** *p* < 0.001.

**Figure 3:**
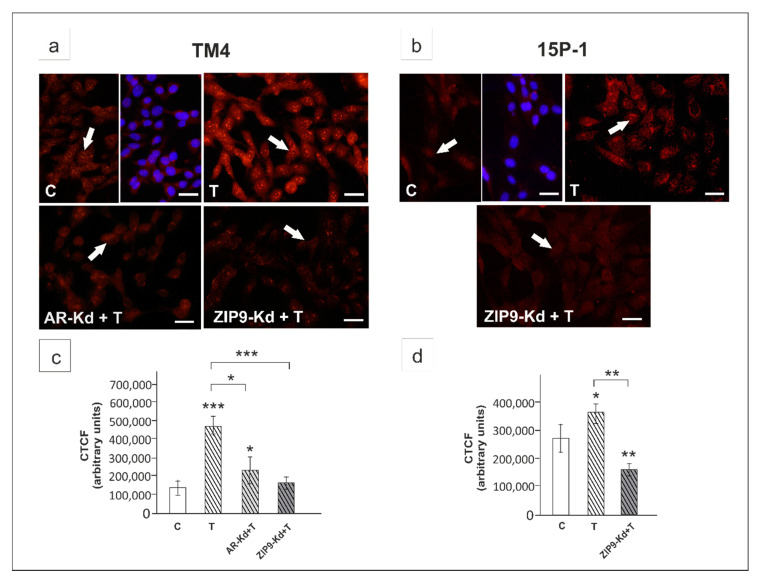
Immunofluorescence analysis of N1ICD expression in TM4 (**a**,**c**) and 15P-1 (**b**,**d**) cells following AR or ZIP9 knockdown. Cells were transfected with transfection reagent alone (C), transfection reagent + 5 × 10^−8^ M AR siRNA (AR-Kd) or ZIP9 siRNA (ZIP9-Kd). After 24 h 10^−8^ M T or vehicle was added to the culture. Cells were fixed after a 24 h incubation. (**a**,**b**) Representative microphotographs of TM4 and 15P-1 cultures. Arrows indicate positive signal. Sertoli cell nuclei were stained with 4′,6-diamidino-2-phenylindole (DAPI; blue). Right panels in controls (C) represent the merged images. Scale bar = 25 µm. (**c**,**d**) The fluorescence intensity was quantified using ImageJ and displayed in corrected total cell fluorescence (CTCF). Histograms represent the mean ± SD. Significant differences from control values are denoted as * *p* < 0.05, ** *p* < 0.01, and *** *p* < 0.001.

Next, to determine the involvement of AR and ZIP9 in the regulation of Notch pathway activity we knocked down each of the receptors using RNAi technique. In TM4 cells, knockdown of either AR or ZIP9 partly abrogated the effect of testosterone on RBP-J activity (Figure 2a) and *Notch1*/N1ICD level (Figure 2c,e) (*p* < 0.05, *p* < 0.01, *p* < 0.001), which suggests that both receptors modulate Notch signaling activity. In *Ar*-silenced cells, weaker immunofluorescence signal of N1ICD was observed mainly in cell nuclei, whereas ZIP9 knockdown caused signal reduction in both nuclei and cytoplasm (Figure 3a) when compared to the control. In 15P-1 cells, *Zip9* silencing fully blocked testosterone-induced Notch pathway activation (*p* < 0.001) (Figure 2b,d,f) and N1ICD signal almost totally disappeared (Figure 3b). Qualitative analyses of N1ICD signal were confirmed using quantitative image analysis (Figure 3c,d).

Further, we tested the effect of pharmacological anti-androgens hydroxyflutamide (HF) and bicalutamide (Bic) on Notch pathway activation in Sertoli cell lines and primary Sertoli cells (PSC). HF is an active metabolite of pure AR antagonist, flutamide, which does not block membrane-initiated androgen actions [12,37,38,39]. In contrast, Bic was demonstrated to inhibit activation of both AR and ZIP9 [40,41].

**Figure 4:**
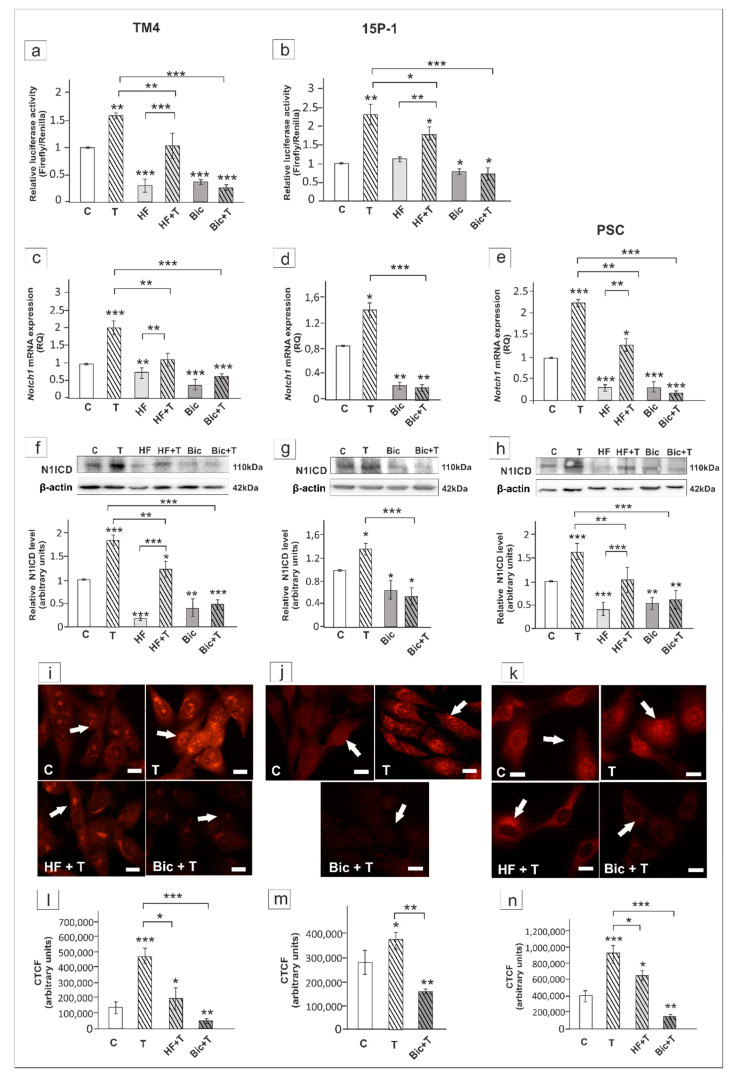
The effect of hydroxyflutamide (HF) and bicalutamide (Bic) on Notch pathway activity, Notch1 mRNA and N1ICD expression in TM4 (**a**,**c**,**f**,**i**,**l**), 15P-1 (**b**,**d**,**g**,**j**,**m**), and primary (PSC; **e**,**h**,**k**,**n**) Sertoli cells. (**a**,**b**) TM4 and 15P-1 cells were transfected with 2 × 10^−12^ M of RBP-J reporter. After 24 h cells were treated with 10^−8^ M testosterone (T), 10^−4^ M hydroxyflutamide (HF), 10^−6^ M bicalutamide (Bic) or vehicle (C). Cells were harvested after a 24 h and the ratio of firefly-derived luminescence over renilla-derived luminescence was determined. Data are means ± SD for a single experiment done in triplicate. (**c**–**k**) Cells were treated 10^−8^ M T, 10^−4^ HF, HF + T, 10^−6^ M Bic, Bic + T or vehicle (C) for 24 h. (**c**–**e**) Relative expression of mRNAs (RQ) was determined using real-time RT-PCR analysis. The expression values of the individual genes were normalized to the mean expression of *Rn18s*, *B2m*, *Gapdh* and *Actb*. (**f**–**h**) Western blot detection of the proteins. The relative level of studied protein was normalized to β-actin. The protein levels within the control group were arbitrarily set at 1. The histograms are the quantitative representation of data (mean ± SD) of three independent experiments, each in triplicate. (**i**–**k**) Immunofluorescence analysis of N1ICD expression. Arrows indicate positive signal. Scale bar = 10 µm. (**l**–**n**) The fluorescence intensity was quantified using ImageJ and displayed in corrected total cell fluorescence (CTCF). Histograms represent the mean ± SD. Significant differences from control values are denoted as * *p* < 0.05, ** *p* < 0.01, and *** *p* < 0.001.

HF partly blocked testosterone effect on RBP-J transcriptional activity in TM4 cells (*p* < 0.01) (Figure 4a) but had no effect in 15P-1 cells (Figure 4b). Bic effectively inhibited testosterone-induced RBP-J activity in both cellular models studied (*p* < 0.001) (Figure 4a,b).

The effect of testosterone on *Notch1*/N1ICD expression was partly suppressed by HF in both TM4 (*p* < 0.01; Figure 4c,f,l) and PSC (*p* < 0.01; Figure 4e,h,n), whereas Bic completely inhibited testosterone-induced *Notch1*/N1ICD expression in all cellular models studied (*p* < 0.001; Figure 4c–h,l–n). Loss of immunofluorescence signal of N1ICD following Bic exposure was seen (Figure 4i–k).

**Figure 5:**
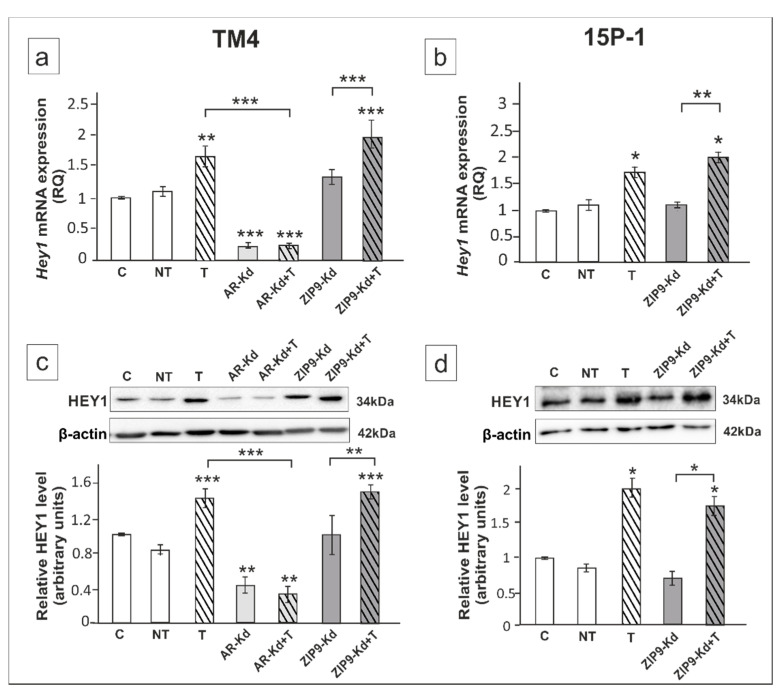
The effect of AR and ZIP9 knockdown on *Hey1* mRNA and HEY1 protein expression in TM4 (**a**,**c**) and 15P-1 (**b**,**d**) Sertoli cells. Cells were treated with transfection reagent alone (C), transfection reagent + 5 × 10^−8^ M non-targeting siRNA (negative control, NT), transfection reagent + 5 × 10^−8^ M AR siRNA (AR-Kd) or ZIP9 siRNA (ZIP9-Kd). After 24 h 10^−8^ M T or vehicle was added to the culture. (**a**,**b**) Relative expression of mRNAs (RQ) was determined using real-time RT-PCR analysis. The expression values of the individual genes were normalized to the mean expression of *Rn18s*, *B2m*, *Gapdh* and *Actb*. (**c**,**d**) Western blot detection of the proteins. The relative level of studied protein was normalized to β-actin. The protein levels within the control group were arbitrarily set at 1. The histograms are the quantitative representation of data (mean ± SD) of three independent experiments, each in triplicate. Significant differences from control values are denoted as * *p* < 0.05, ** *p* < 0.01, and *** *p* < 0.001.

Finally, we examined the effect of androgen receptor signaling inhibition on the expression of Notch pathway effector genes *Hes1* and *Hey1*. AR knockdown in TM4 cells abolished the effect of testosterone on *Hey1* mRNA and protein expression (*p* < 0.001), whereas ZIP9 knockdown had no effect (Figure 5a–d).

**Figure 6:**
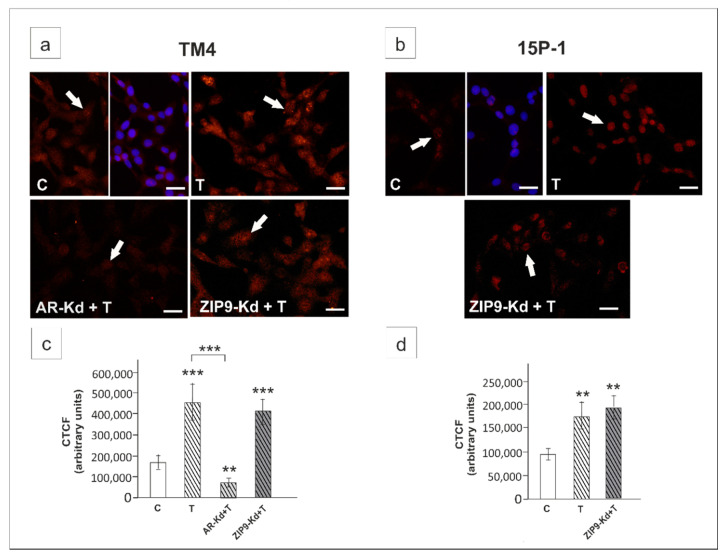
Immunofluorescence analysis of HEY1 expression in TM4 (**a**,**c**) and 15P-1 (**b**,**d**) cells following AR or ZIP9 knockdown. Cells were transfected with transfection reagent alone (C), transfection reagent + 5 × 10^−8^ M AR siRNA (AR-Kd) or ZIP9 siRNA (ZIP9-Kd). After 24 h 10^−8^ M T or vehicle was added to the culture. Cells were fixed after a 24 h incubation. (**a**,**b**) Representative microphotographs of TM4 and 15P-1 cultures. Arrows indicate positive signal. Sertoli cell nuclei were stained with DAPI (blue). Right panels in controls (C) represent the merged images. Scale bar = 25 µm. (**c**,**d**) The fluorescence intensity was quantified using ImageJ and displayed in corrected total cell fluorescence (CTCF). Histograms represent the mean ± SD. Significant differences from control values are denoted as ** *p* < 0.01, and *** *p* < 0.001.

Immunofluorescence revealed decreased signal only in *Ar*-silenced cells (Figure 6a,c). In 15P-1 cells testosterone-stimulated expression of HEY1 was not affected by *Zip9* silencing (Figure 4b,d).

**Figure 7:**
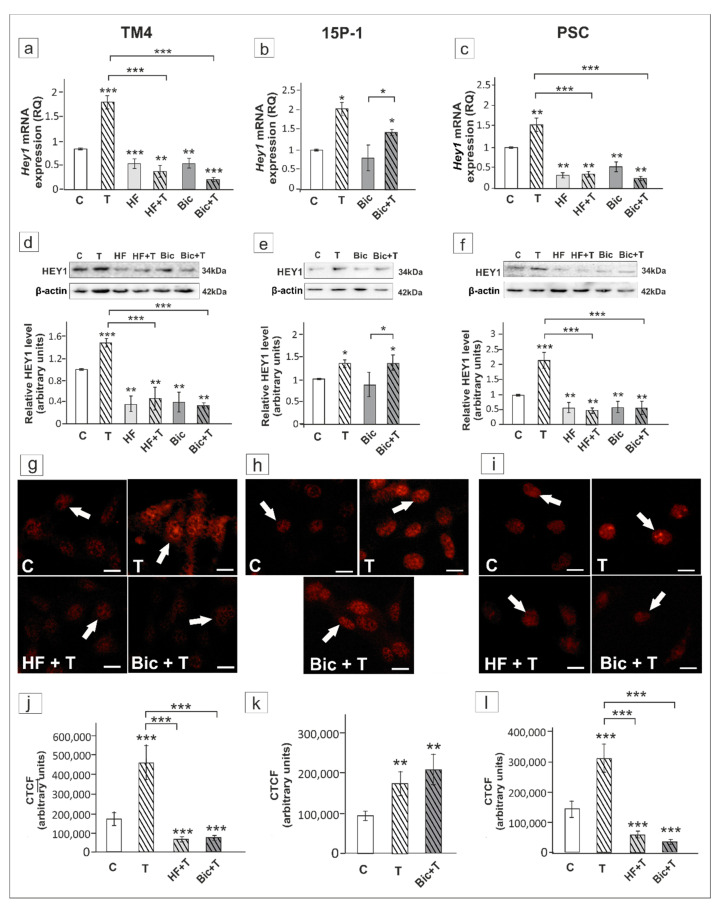
The effect of hydroxyflutamide (HF) and bicalutamide (Bic) on *Hey1* mRNA and HEY1 protein expression in TM4 (**a**,**d**,**g**,**j**), 15P-1 (**b**,**e**,**h**,**k**), and primary (PSC; **c**,**f**,**j**,**l**) Sertoli cells. Cells were treated with 10^−8^ M testosterone (T), 10^−4^ HF, HF + T, 10^−6^ M Bic, Bic + T or vehicle (C) for 24 h. (**a**–**c**) Relative expression of mRNAs (RQ) was determined using real-time RT-PCR analysis. The expression values of the individual genes were normalized to the mean expression of *Rn18s*, *B2m*, *Gapdh* and *Actb*. (**d**–**f**) Western blot detection of the proteins. The relative level of studied protein was normalized to β-actin. The protein levels within the control group were arbitrarily set at 1. The histograms are the quantitative representation of data (mean ± SD) of three independent experiments, each in triplicate. (**g**–**i**) Immunofluorescence analysis of HEY1 expression. Arrows indicate positive signal. Scale bar = 10 µm. (**j**–**l**) The fluorescence intensity was quantified using ImageJ and displayed in corrected total cell fluorescence (CTCF). Histograms represent the mean ± SD. Significant differences from control values are denoted as * *p* < 0.05, ** *p* < 0.01, and *** *p* < 0.001.

In TM4 and PSCs, HF completely abolished the effect of testosterone on *Hey1* expression (*p* < 0.001). Similar effect was observed for Bic (*p* < 0.01) (Figure 7a,c,d,f). Immunofluorescence signal was clearly reduced in both HF and Bic treated cells (Figure 7g,i,j,l). In contrast, Bic had no effect on basal and testosterone-stimulated *Hey1* expression in 15P-1 cells, which was also demonstrated by immunofluorescence (Figure 7b,e,h,k). This indicates that AR-mediated signaling is involved in the regulation of *Hey1* in Sertoli cells, whereas ZIP9 is not implicated.

**Figure 8:**
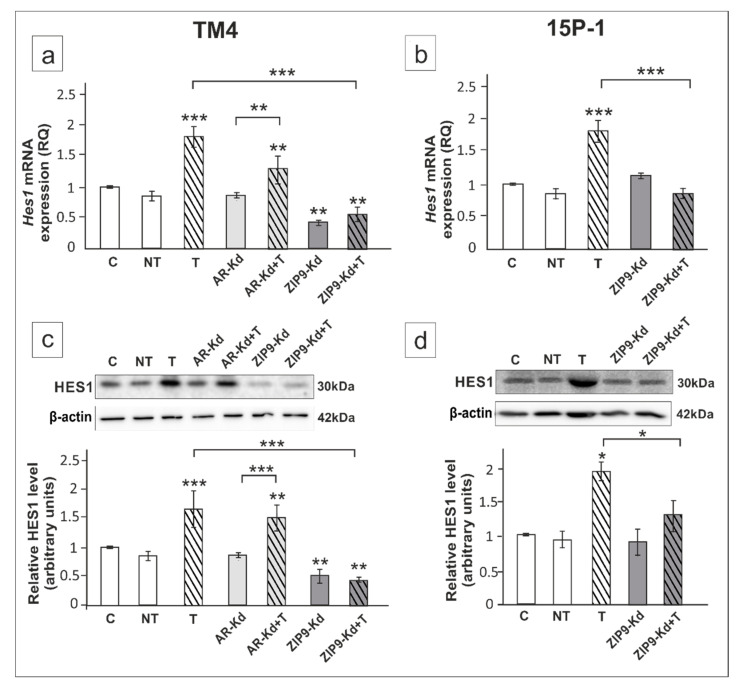
The effect of AR and ZIP9 knockdown on *Hes1* mRNA and HES1 protein expression in TM4 (**a**,**c**) and 15P-1 (**b**,**d**) Sertoli cells. Cells were treated with transfection reagent alone (C), transfection reagent + 5 × 10^−8^ M non-targeting siRNA (negative control, NT), transfection reagent + 5 × 10^−8^ M AR siRNA (AR-Kd) or ZIP9 siRNA (ZIP9-Kd). After 24 h 10^−8^ M T or vehicle was added to the culture. (**a**,**b**) Relative expression of mRNAs (RQ) was determined using real-time RT-PCR analysis. The expression values of the individual genes were normalized to the mean expression of *Rn18s*, *B2m*, *Gapdh* and *Actb*. (**c**,**d**) Western blot detection of the proteins. The relative level of studied protein was normalized to β-actin. The protein levels within the control group were arbitrarily set at 1. The histograms are the quantitative representation of data (mean ± SD) of three independent experiments, each in triplicate. Significant differences from control values are denoted as * *p* < 0.05, ** *p* < 0.01, and *** *p* < 0.001.

In TM4 cells *Ar* silencing had no effect on *Hes1* expression (Figure 8a,c), while ZIP9 knockdown significantly reduced testosterone-stimulated *Hes1* expression in both TM4 and 15P-1 cells (*p* < 0.05, *p* < 0.001) (Figure 8a–d).

**Figure 9:**
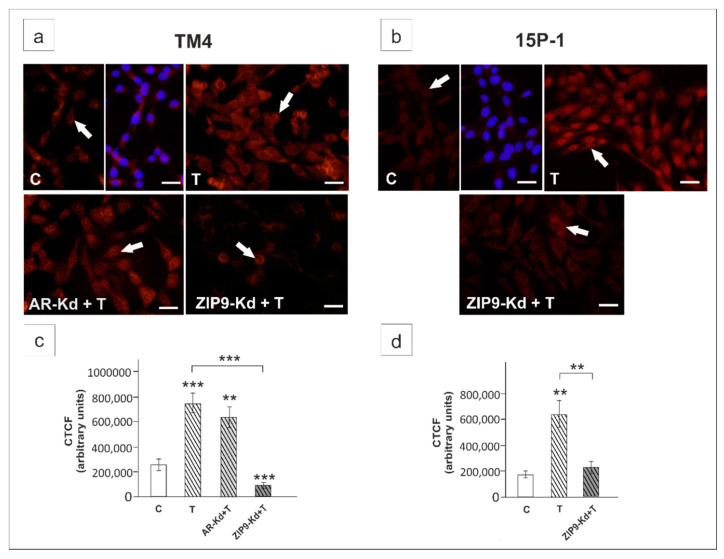
Immunofluorescence analysis of HES1 expression in TM4 (**a**,**c**) and 15P-1 (**b**,**d**) cells following AR or ZIP9 knockdown. Cells were transfected with transfection reagent alone (C), transfection reagent + 5 × 10^−8^ M AR siRNA (AR-Kd) or ZIP9 siRNA (ZIP9-Kd). After 24 h 10^−8^ M T or vehicle was added to the culture. Cells were fixed after a 24 h incubation. (**a**,**b**) Representative microphotographs of TM4 and 15P-1 cultures. Arrows indicate positive signal. Sertoli cell nuclei were stained with DAPI (blue). Right panels in controls (C) represent the merged images. Scale bar = 25 µm. (**c**,**d**) The fluorescence intensity was quantified using ImageJ and displayed in corrected total cell fluorescence (CTCF). Histograms represent the mean ± SD. Significant differences from control values are denoted as ** *p* < 0.01, and *** *p* < 0.001.

In testosterone-exposed cells immunofluorescence signal of HES1 was localized to both cell nuclei and cytoplasm (Figure 9a,b). In *Ar*-silenced TM4 cells signal was maintained mainly in cell nuclei, whereas ZIP9 knockdown resulted in marked reduction of signal intensity in cell cytoplasm, and loss of the signal in most cell nuclei of both TM4 and 15P-1 (Figure 9a,b). Qualitative analyses of signals were confirmed using quantitative analysis of fluorescence intensity (Figure 9c,d).

**Figure 10:**
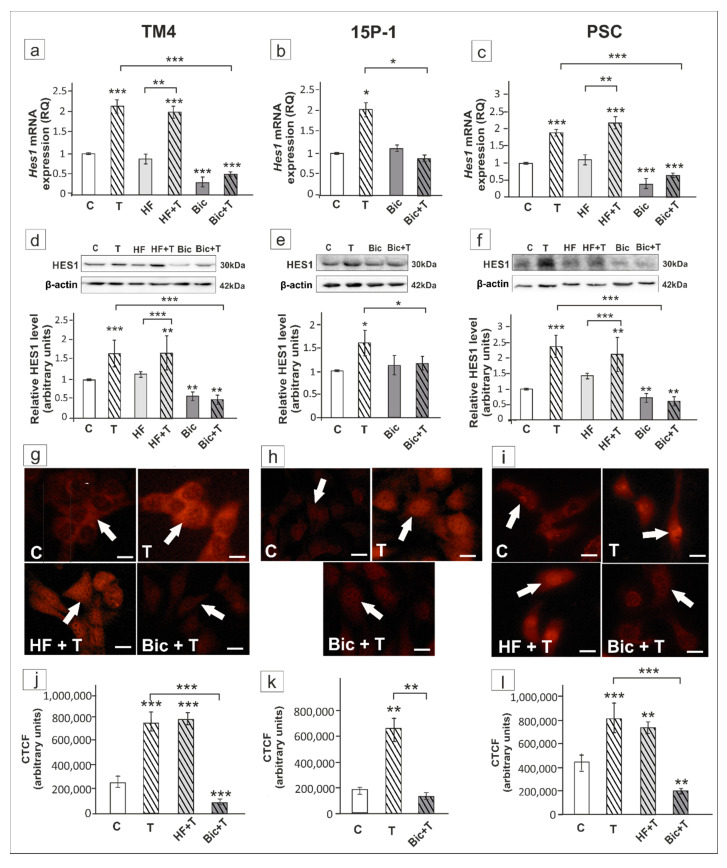
The effect of hydroxyflutamide (HF) and bicalutamide (Bic) on *Hes1* mRNA and HES1 protein expression in TM4 (**a**,**d**,**g**,**j**), 15P-1 (**b**,**e**,**h**,**k**), and primary (PSC; **c**,**f**,**i**,**l**) Sertoli cells. Cells were treated with 10^−8^ M testosterone (T), 10^−4^ HF, HF + T, 10^−6^ M Bic, Bic + T or vehicle (C) for 24 h. (**a**–**c**) Relative expression of mRNAs (RQ) was determined using real-time RT-PCR analysis. The expression values of the individual genes were normalized to the mean expression of *Rn18s*, *B2m*, *Gapdh* and *Actb*. (**d**–**f**) Western blot detection of the proteins. The relative level of studied protein was normalized to β-actin. The protein levels within the control group were arbitrarily set at 1. The histograms are the quantitative representation of data (mean ± SD) of three independent experiments, each in triplicate. (**g**–**i**) Immunofluorescence analysis of HES1 expression. Arrows indicate positive signal. Scale bar = 10 µm. (**j**–**l**) The fluorescence intensity was quantified using ImageJ and displayed in corrected total cell fluorescence (CTCF). Histograms represent the mean ± SD. Significant differences from control values are denoted as * *p* < 0.05, ** *p* < 0.01, and *** *p* < 0.001.

In testosterone-treated TM4 and PSC cells the expression of *Hes1*/HES1 was not affected by HF (Figure 10a,c,d,f), whereas Bic inhibited the effect of testosterone on *Hes1* mRNA and HES1 protein expression in all cellular models (*p* < 0.01, *p* < 0.001) (Figure 10a–f). In TM4 and PSC cells exposed to HF immunofluorescence signal was detected mainly in cell nuclei (Figure 10g,i). In Bic treated cells loss of nuclear HES1 staining was found (Figure 10g–i). Qualitative analysis revealed significant reduction of fluorescence intensity following Bic exposure when compared to cultures treated with testosterone alone (*p* < 0.01, *p* < 0.001) (Figure 10j–l). Taken together, these results suggest that *Hes1* is regulated via ZIP9-dependent signaling in rodent Sertoli cells.

## 3. Discussion

In the present paper we show that activity of Notch signaling is regulated by testosterone acting via both AR and ZIP9 receptors, thus providing evidence that a crosstalk between Notch and androgen pathways exists in Sertoli cells. Herein, we found that testosterone is a potent stimulator not only of Notch1 receptor expression, but also its activation, as demonstrated by increased N1ICD level, increased nuclear accumulation of N1ICD and enhanced RBP-J transcriptional activity. The stimulatory effect of androgens on Notch1 was previously described in myogenic progenitor cells during pubertal development [42]. In contrary, in ventral prostate of orchiectomized rats, inhibitory action of androgen on Notch1 signaling was reported [29]. Therefore, the effect of androgens on Notch1 signaling clearly depends on cellular context. Previous studies showed that epigenetic factors and availability of cofactors influence the AR-regulated transcriptome and finally determine the final response of the cell [43,44]. Moreover, several papers reported that testosterone binding to ZIP9 activates different G proteins and various downstream signaling events in cell type-specific manner [8,12,45,46].

In TM4 cells, *Ar*-silencing only partly blocked the effect of testosterone on Notch1 pathway activity, suggesting that AR-mediated action is not the only mechanism involved in testosterone effect on Notch pathway. In agreement, in both TM4 and PCS cells, HF (AR antagonist) was unable to totally block the effect of testosterone. An increase in Notch signaling activity following testosterone exposure was significantly lower in HF-treated cells than in vehicle-treated cells. Moreover, the stimulatory effect of testosterone on Notch signaling was also observed in 15P-1 cells, in which we detected no AR expression. Taken together, these results suggested that other receptor(s) besides the AR may be involved in the regulation of Notch pathway by testosterone.

RT-PCR, western blotting and immunofluorescence analyses confirmed that all cellular models used herein express ZIP9. Both, ZIP9 knockdown or treatment of 15P-1 cells with Bic (antagonist of AR and ZIP9) abolished testosterone-induced upregulation of *Notch1*/N1ICD expression and Notch pathway activity. This led us to the conclusion that both AR and ZIP9 are involved in testosterone-mediated increase in Notch signaling in Sertoli cells.

The activity of canonical Notch pathway in Sertoli cells is manifested by enhanced expression of HES1 and HEY1 transcriptional repressors [9,17,21]. Accordingly, we found that testosterone-induced Notch signaling activity was followed by upregulation of *Hey1* and *Hes1* mRNA and protein. However, androgen receptors’ silencing experiments and antiandrogen exposures provided evidence that different androgen signaling pathways are involved in the control of each effector gene. In TM4 and PSC AR knockdown or inhibition, respectively, abolished the effect of testosterone on HEY1 expression, indicating that HEY1 is regulated by AR-dependent pathway. In prostate cancer cells HEY1 functions as a corepressor for AF1 in the AR, inhibiting transcription from androgen-dependent target genes [47]. Although such interaction has not been confirmed in Sertoli cells yet, HEY1 may be considered as a factor involved in AR-controlled cellular responses. It is possible that HEY1 upregulation in response to AR activation may limit AR-regulated transcription, serving as negative feedback regulatory mechanism in AR-expressing cells.

*Zip9* silencing in TM4 cells was ineffective in blocking testosterone action on HEY1 expression or localization, which suggested that ZIP9 was not involved in its regulation. This was confirmed in 15P-1 cells, where testosterone exposure increased HEY1, but ZIP9 knockdown or exposure to Bic were not able to prevent the action of testosterone. Since AR was undetectable in 15P-1 cells, our results suggest that some other AR- and ZIP9-independent mechanism is involved in HEY1 regulation by androgen in AR-negative Sertoli cells. In recent years TRPM8 (transient receptor potential cation channel subfamily M member 8) and GPRC6A (G protein-coupled receptor class C group 6 member A) proteins were demonstrated to mediate the action of androgens in prostate cancer cells [48,49,50]. Both proteins are localized also in rodent Sertoli cells [51,52], but their role as potential androgen receptors in these cells remains to be investigated.

Reduction of AR signaling by siRNA or HF exposure was unable to prevent stimulatory effect of testosterone on *Hes1* expression in TM4 and PSC, whereas ZIP9 knockdown or inhibition by Bic effectively blocked this effect, demonstrating that ZIP9 is involved in the regulation of HES1 expression. This is in agreement with our previous observations that in vivo AR blockade by flutamide did not attenuate the immunoexpression of HES1 in rat Sertoli cells, whereas testosterone reduction led to a decrease of HES1 immunostaining intensity measured with densitometry [33]. Interestingly, although strong immunofluorescence signal was maintained in cell nuclei following AR knockdown or inhibition, signal intensity was diminished in cell cytoplasm. This may suggest that despite no effect on HES1 expression level, AR-mediated signaling influence subcellular distribution of HES1 protein, regulating its nuclear transport. On the other hand, AR was required for FOXA1-enhanced Notch pathway activation in endometrial cancer cells, increasing Notch1 and HES1 expression [53]. Nevertheless, in Sertoli cells, the expression of two effector proteins HEY1 and HES1 is regulated by androgens via different signaling pathways. Diverse HEY1 and HES1 regulation by dihydrotestosterone was earlier observed in R1 castrate-resistant prostate cancer cell line, however the mechanism was not investigated in that study [54]. In light of the data, further studies are required to understand how AR and ZIP9 differentially regulate *Hes1* and *Hey1* expression by activation of transcriptional activity of RBP-J in Sertoli cells. Based on the recent findings by Clocchiatti et al. [55], who demonstrated physical association between AR and RBP-J in primary human dermal fibroblasts, it is likely that both proteins are constituents of a common, ligand-dependent complex, which regulates transcription of target genes. To date, evidence for direct interaction between ZIP9 and NICD is lacking. However, CREB consensus motifs were found in association with numerous RBP-J sites in murine T-cells [56], which may indicate a possible interaction with CREB-mediated signaling pathways, such as ZIP9 signaling. Currently, it is becoming evident that in addition to the DNA sequence, other factors, such as protein-protein interactions, may play a role in RBP-J associations with chromatin. In consequence, interactions with different transcription factors may modulate activities of RBP-J, and thus Notch signaling could be diversified through differential DNA targeting [57].

Finally, it should be noted that cell culture models used in the present study allow us to obtain precise insight into the interactions of particular signaling pathways in Sertoli cells, however further research using AR and ZIP9 knock-out mouse models are necessary to confirm these relations in the environment of seminiferous epithelium in vivo.

Taken together, testosterone regulates Notch signaling in Sertoli cells acting at various levels of this pathway. Androgen signaling through both AR and ZIP9 receptors controls not only the expression and activation of Notch1, RBP-J transcriptional activity, the expression of Notch target genes, and distribution of the effector proteins within the cells, as demonstrated herein, but also the expression of Notch ligands in both Sertoli and germ cells, as we reported previously [33]. Another possible androgen target is γ-secretase and the process of Notch receptor cleavage. It was demonstrated that presenilin 1 (PS1), an enzymatic unit of γ-secretase, is regulated by testosterone in the cerebral cortex of male mice in age-dependent manner [58] and its expression is altered in hippocampus of hypogonadal male mice [59]. In addition, androgens may act on intracellular regulators of Notch signaling. For instance, androgens up-regulate transcription of Notch inhibitor, Numb, in prostate cancer cells [60]. Finally, it cannot be excluded that, at least to some extent, androgens may modulate *Hes1* or *Hey1* expression in Notch-independent manner, as it was described for other signaling pathways [61]. Whether any of these mechanisms are also involved in androgen effect on Notch signaling in Sertoli cells needs further consideration.

## 4. Materials and Methods

### 4.1. Cell Line Cultures and Treatments

The mouse Sertoli cell lines TM4 (Cat no. CRL-1715) and 15P-1 (Cat no. CRL-2618) were purchased from American Type Culture Collection (ATCC, Manassas, VA, USA) and were maintained under standard conditions. Properties of both cell lines were evaluated according to cell line authentication recommendations of the Global Bioresource Center (ATCC). Microscopic observation, analysis of proliferation and viability and detection cell-specific markers expression (androgen binding protein (*Abp*), Desert Hedgehog (*Dhh),* SRY-box 9 (*Sox9*), sulphated glycoprotein-2 (*Sgp-2*), GATA binding protein 4 (*Gata4*); Institute of Biochemistry and Biophysics, Polish Academy of Sciences, Warsaw, Poland; Table S1) were performed. Cells were seeded directly into cell culture plates or on coverslips. Before each experiment, cells were serum starved for 24 h. The same cell concentration (0.5 × 10^6^/cm^2^) was used in all experiments, so the ratio of T concentration and initial cell number was constant between experiments.

Cells were treated with 10^−8^ M to 10^−6^ M testosterone (T, Cat no. 86500; Sigma-Aldrich, St. Louis, MO, USA) or anti-androgens 10^−4^ M hydroxyflutamide (HF; Cat no. H4166; Sigma-Aldrich) or 10^−6^ M bicalutamide (Bic) (Cat no. B9061; Sigma-Aldrich) alone or with the addition of 10**^−^**^8^ M T for 24 h. Control cells were incubated in the presence of the vehicle (0.01% dimethyl sulfoxide, DMSO).

### 4.2. Transfection of TM4 or 15P-1 Cells with siRNA Duplexes

To knockdown AR or ZIP9 in Sertoli cells by RNAi, TM4 or 15P-1, cells were seeded at 0.1 × 10^5^ cells/cm^2^ in 6-well plates [9]. Next, the cells were transfected with Silencer™ Select siRNAs (AR-specific siRNA assay ID: s62547, ZIP9-specific siRNA assay ID: s116149; Thermo Fisher Scientific, Waltham, MA, USA) using jetPrime Transfection Reagent (Polyplus-Transfection S.A., Bioparc, Illkirch, France) in serum-free Opti-MEM (Cat no. 11058021; Life Technologies, Gaithersburg, MD, USA), according to the manufacturer’s instructions. Negative control cells were treated with jetPrime Transfection Reagent alone or jetPrime Transfection Reagent plus Silencer™ Select Negative Control No. 1 (non-targeting siRNA; Cat no. 4404020; Thermo Fisher Scientific). Positive control was performed using Silencer™ Select GAPDH Positive Control siRNA (Cat no. 4390849; Thermo Fisher Scientific). After 24 h, cells were washed twice to remove silencing duplexes and transfection medium. Cells were treated with 10^−8^ M T or a vehicle for 24 h. Transfection efficiencies were in the range of 87 ± 2% for AR siRNA and 73 ± 5% for ZIP9 siRNA. No cytotoxic effect of used concentrations of siRNAs was detected (CellTiter-Glo^®^ Luminescent Cell Viability Assay, Cat no. G7570; Promega, Madison, WI, USA).

### 4.3. Reporter Assay

RBP-J reporter assay was performed using Cignal RBP-J Pathway Reporter Assay (Cat No. 336841; Qiagen, Hilden, Germany) according to manufacturer’s instruction. In the first experiment, TM4 and 15P-1 cells were transfected with RBP-J reporter (a mixture of an inducible RBP-J responsive firefly luciferase reporter and constitutively expressing Renilla construct) and AR siRNA or ZIP9 siRNA. After 16 h cells were treated with T or vehicle. In the second experiment, cells were transfected with RBP-J reporter and after 24 h treated with T, HF or Bic. 

Luciferase activity was measured after 24 h using Dual-Glo Luciferase Assay System (Promega) with TECAN Infinite M200 Pro luminometer (Tecan; Männedorf, Switzerland). Firefly luciferase experimental reporter was normalized to Renilla luciferase activity to control transfection efficiency. 

Negative controls were performed as follows: (i) RBP-J reporter and non-targeting siRNA; (ii) a mixture of non-inducible firefly luciferase reporter and constitutively expressing Renilla construct (Cignal Negative Control); (iii) Cignal Negative Control and AR siRNA or ZIP9 siRNA; (iv) Cignal Negative Control and non-targeting siRNA. As a positive control a mixture of a constitutively expressing GFP construct, constitutively expressing firefly luciferase construct, and constitutively expressing Renilla luciferase construct was used (not shown).

### 4.4. Primary Sertoli Cell (PSC) Culture and Treatments

Sertoli cells were isolated from 20-day-old rat testes according to previously described protocol [62]. Sertoli cells from 20-day-old rat are differentiated and have almost negligible contamination with somatic and germ cells. Primary cells were plated onto Matrigel- (Cat no. 354234; BD Biosciences, San Jose, CA, USA) coated 12-well plates at 0.5 × 10^6^ cells/cm^2^ and incubated in serum-free Dulbecco’s Modified Eagle Medium (DMEM) supplemented with growth factors and an antibiotic in a humidified atmosphere of 95% air and 5% CO_2_ (vol/vol) at 35 °C. At 48 h after plating, cultures were treated with a hypotonic buffer (20 mM Tris pH 7.4 at 22 °C) to lyse contaminating germ cells and obtaining primary Sertoli cell cultures with 98% purity [63,64]. Next, primary Sertoli cells were treated with 10^−8^ M T, 10^−4^ M HF, or 10^−6^ M Bic alone or with T for 24 h. Control cells were incubated in the presence of the vehicle (0.01% DMSO).

### 4.5. RNA Isolation, Reverse Transcription and Real-Time Quantitative RT-PCR

Total RNA was extracted from cells with TRIzol^®^ reagent (Cat no. 15596026; Life Technologies) according to the manufacturer’s instructions. Contaminating DNA and DNase was removed with TURBO DNA-free™ Kit (Cat no. AM1907; Ambion, Austin, TX, USA) according to the manufacturer’s instructions. The yield and quality of the RNA were assessed by determining the A260:A280 ratio (NanoDrop ND2000 Spectrophotometer, Thermo Fisher Scientific) and by electrophoresis. An A260:280 ratio not lower than 1.9 was accepted for cDNA synthesis.

High-Capacity cDNA Reverse Transcription Kit (Cat no. 4368814; Applied Biosystems, Carlsbad, CA, USA) was used to obtain total cDNA. Reactions for each RNA sample were run in the absence of RT to assess genomic DNA contamination. Real-time RT-PCR analyses were performed with the StepOne Real-time PCR system (Applied Biosystems) with the cDNA templates, primers (Institute of Biochemistry and Biophysics, Polish Academy of Sciences) listed in Table 1 and SYBR Green master mix (Cat no. 4309155; Applied Biosystems), as described previously [9]. 

Amplification efficiency [65], was between 97% and 104%. Melting curve analysis and subsequent agarose gel electrophoresis were used to confirm amplification specificity. In all real-time RT-PCR reactions, a negative control corresponding to RT reaction without the reverse transcriptase enzyme and a blank sample were carried out. mRNA expressions were normalized to the mean expression of reference genes *Rn18s*, *B2m*, *Actb* and *Gapdh* mRNA (relative quantification, RQ = 1) with the use of the 2^−ΔΔCt^ method [66]. 

### 4.6. Western Blot Analysis 

Lysates were obtained by sample sonification with a cold Tris/EDTA buffer (50 mM Tris, 1 mM EDTA, pH 7.5) supplemented with protease inhibitors (Cat no. P8340; Sigma-Aldrich). Samples were resolved by sodium dodecyl sulfate polyacrylamide gel electrophoresis (SDS-PAGE) under reducing conditions, transferred to polyvinylidene difluoride membranes (Sigma-Aldrich) and analyzed by immunoblotting with the respective primary antibodies as previously reported in detail [63]. Briefly, nonspecific binding sites were blocked with 5% (*wt/v*) non-fat dry milk containing 0.1% (*v/v*) Tween20. Next, the membrane was incubated with the respective primary antibody (Table 2) at 4 °C overnight, followed by a horseradish peroxidase-conjugated secondary antibody (1:3000; Cat no. PI-1000-1; PI-2000-1; Vector Labs., Burlingame, CA, USA) for 1 h at room temperature. 

Proteins were detected by chemiluminescence and images were captured with a ChemiDocTM XRS+ System (Bio–Rad Labs., München, Germany). All immunoblots were stripped and reprobed with an antibody against β-actin (Table 2). The molecular weights of target proteins were estimated by reference to standard proteins (Cat no. 1610397; Bio–Rad Labs.). To obtain semi-quantitative results, immunoblots were analyzed using the ImageLab software (Bio–Rad Labs.). Each data point was normalized against its corresponding actin data point.

### 4.7. Immunofluorescence

Immunofluorescence labeling was performed on cells seeded on coverslips and treated as described in Section 4.1, Section 4.2 and Section 4.4. The cells were washed with phosphate buffered saline (PBS), fixed with methanol–acetone and immunostained as described [67]. Nonspecific binding sites were blocked with 10% normal goat serum (Cat no. G9023; Sigma-Aldrich) for 20 min. Cells were incubated in the presence of primary antibodies (Table 2) at 4 °C overnight. Next, Cy3-cojugated goat anti-Rabbit IgG (1:200; Cat no. A10520; Thermo Fisher Scientific) was applied for 60 min. Coverslips were mounted with Vectashield mounting medium (Cat no. H-1500; Vector Labs.) with 4′,6-diamidino-2-phenylindole (DAPI) and examined with epifluorescence microscope Nikon Eclipse Ni (Nikon Instech Co., Japan). No background fluorescence was observed in the negative controls, where the primary antibody was omitted (not shown). The fluorescent images acquired with a 40× objective were analyzed using ImageJ software (National Institutes of Health, Bethesda, MD). An outline was drawn around each cell (100 cells per sample) and the area, integrated density and mean gray value were measured along with adjacent background readings. Next, corrected total cell fluorescence (CTCF) = integrated density (area of selected cell × mean fluorescence of background readings) was calculated as described previously [68].

### 4.8. Statistical Analysis

Each variable was tested using the Shapiro-Wilk *W*-test for normality. The homogeneity of variance was assessed with Levene’s test. Statistical differences in relative luciferase activity, mRNA expression levels, as well as differences in protein expression levels were assessed using one-way ANOVA, followed by Tukey’s post hoc comparison test. Statistical analyses were performed on raw data using Statistica 10 software (StatSoft Inc., Tulsa, OK, USA). Data were presented as means ± SD. Data were considered statistically significant at * *p* < 0.05, ** *p* < 0.01, *** *p* < 0.001.

## 5. Conclusions

**Figure 11:**
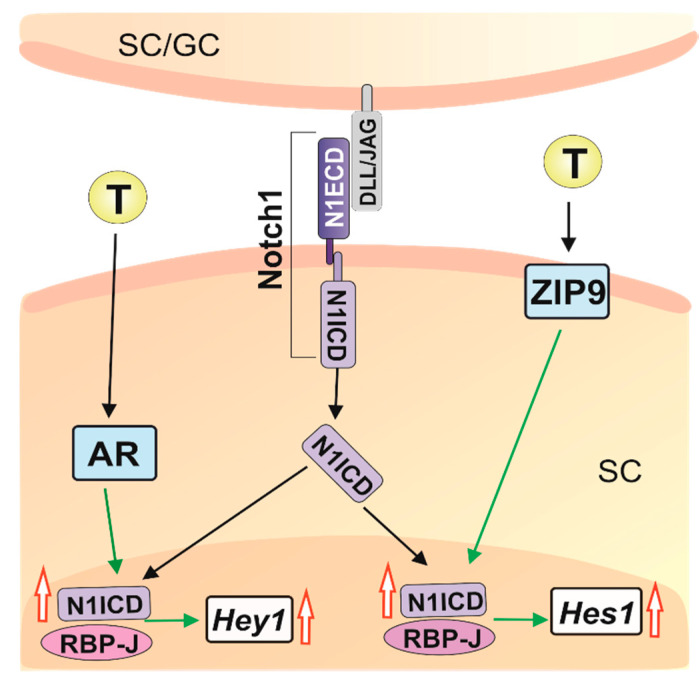
Schematic representation of the crosstalk between androgen and Notch signaling pathways in rodent Sertoli cells. Notch signaling in Sertoli cells may be induced by Delta-like or Jagged ligands expressed either in Sertoli or germ cells. In Sertoli cells, the action of testosterone via both AR and ZIP9 increases Notch1/N1ICD expression and transcriptional activity of RBP-J. AR signaling is involved in the up-regulation of *Hey1* expression, whereas ZIP9-mediated pathway controls *Hes1* expression. Green arrows indicate interactions revealed in the present study. Black arrows indicate established steps of androgen and Notch signaling pathways. Red open arrows—up-regulation. AR—nuclear androgen receptor; GC—germ cells; DLL/JAG—Delta-like/Jagged ligands; N1ICD—Notch1 intracellular domain; RBP-J—Recombination signal binding protein; SC—Sertoli cells; T—Testosterone; ZIP9—Zrt- and Irt-like protein 9.

Our findings demonstrate that androgens influence activity of Notch pathway in rodent Sertoli cells. For the first time, we provided evidence for the role of membrane androgen receptor ZIP9 in the control of Notch pathway. Activation of AR- and ZIP9-mediated signaling produce different but complementary effects on Notch effector gene expression, pointing to cooperation of classical and non-classical androgen signaling pathways in controlling Sertoli cell function (Figure 11). The importance of Notch pathway in Sertoli cells for the regulation of spermatogenesis has just begun to be elucidated and the functional role of androgen-Notch signaling crosstalk in seminiferous epithelium requires further investigation.

## Figures and Tables

**Table 1:** Sequences of forward and reverse primers**.**

Gene	Forward Primer	Reverse Primer
**Mouse**		
*Actb*	CTGGAACGGTGAAGGTGACA	AAGGGGACTTCCTGTAACAATGCA
*B2m*	TTCTGGTGCTTGTCTCACTCA	CAGTATGTTCGGCTTCCCATTC
*Gapdh*	GGAGATTGTTGCCATCAACG	GGAGATTGTTGCCATCAACG
*Hes1*	ACCTTCCAGTGGCTCCTC	TTTAGTGTCCGTCAGAAGAGAG
*Hey1*	GCCGAAGTTGCCCGTTATCTG	GCCGAAGTTGCCCGTTATCTG
*Notch1*	GATGCCACCTGAACAACTGC	TGACAACAGCAACAGCAAGG
*Rn18S*	CTCTGGTTGCTCTGTGCAGT	GGCTCCTTGTAGGGGTTCTC
**Rat**		
*Actb*	AAGTACCCCATTGAACACGG	ATCACAATGCCAGTGGTACG
*B2m*	GGACTGGTCTTTCTATATCCTGGC	GATCACATGTCTCGATCCCAGTAG
*Gapdh*	GGAGATTGTTGCCATCAACG	CACAATGCCAAAGTTGTCA
*Hes1*	GGCAGGCGCACCCCGCCTTG	GCAGCCAGGCTGGAGAGGCT
*Hey1*	AAAGACGGAGAGGCATCATCG	GCAGTGTGCAGCATTTTCAGG
*Notch1*	GCAGCCACAGAACTTACAAATCCAG	TAAATGCCTCTGGAATGTGGGTGAT
*Rn18S*	GCCGCGGTAATTCCAGCTCCA	CCCGCCCGCTCCCAAGATC

**Table 2:** Details of primary antibodies used for Western blot and immunofluorescence**.**

Antibody	Host Species	Vendor	Cat. Number	Dilution
Anti-actin	Mouse	Sigma-Aldrich	A2228	1:3000 (WB)
Anti-HES1	Rabbit	Thermo Fisher	PA5-28802	1:1000 (WB); 1:100 (IF)
Anti-HEY1	Rabbit	Thermo Fisher	PA5-40553	1:2000 (WB), 1:100 (IF)
Anti-N1ICD	Rabbit	Abcam	ab8925	1:1000 (WB), 1:200 (IF)

IF—immunofluorescence; WB—Western blot.

## Supplementary Materials

Supplementary materials can be found at https://www.mdpi.com/1422-0067/21/21/8275/s1, Table S1: Sequences of forward and reverse primers used for detection of Sertoli cell-specific markers.

## Abbreviations

AR	Nuclear androgen receptor
ATF-1	Activating transcription factor 1
Bic	Bicalutamide
CREB	cAMP-response element binding protein
DAPI	4′,6-diamidino-2-phenylindole
DMEM	Dulbecco′s Modified Eagle Medium
DMSO	Dimethyl sulfoxide
EDTA	Ethylenediaminetetraacetic acid
ERK 1/2	Extracellular signal-regulated protein kinase 1/2
FOXA1	Forkhead box protein A1
GPRC6A	G protein-coupled receptor class C group 6 member A
*Hes1*/HES1	Hairy/enhancer of split 1
*Hey1*/HEY1	Hes-related with YRPW motif 1
HF	Hydroxyflutamide
N1ICD	Notch1 intracellular domain
PSC	Primary Sertoli cells
RBP-J	Recombination signal binding protein
RT-PCR	Reverse-transcriptase polymerase chain reaction
SDS-PAGE	Sodium dodecyl sulfate – poliacrylamide gel electrophoresis
TRPM8	Transient receptor potential cation channel subfamily M member 8
ZIP9	Zrt- and Irt-like protein 9
