# Supplementary material for: Crosstalk between Androgen-ZIP9 Signaling and Notch Pathway in Rodent Sertoli Cells
Source: Int J Mol Sci. 2020 Nov 5;21(21):8275. doi: 10.3390/ijms21218275 (PMC7663815; doi:10.3390/ijms21218275)
Supplement: Supplementary file 1 [file ijms-21-08275-s001.pdf]

International Journal of
Molecular Sciences

MDPI

Table S1. Sequences of forward and reverse primers used for detection of Sertoli cell-specific markers

Gene	Forward primer	Reverse primer
Abp	AGCCAGCCGGACATGTTG	TGGTGAGGAGGCAATTGGTT
Dhh	AGTAGGTTCCAGGTTCCCCA	GGTTGCGGGACTCGTAGTAG
Gata4	CCGCAAGGCATCTGGCAAA	CGGGAGGTAGAGGCAGGA
Sgp2	GGACAATGGCATGGTCCTGGGAGAG	GGCTTACACTCTTCCCAGAGGGCCA
Sox9	GCTCAGTTCACCGATGTCCA	CCAGCAAGAACAAGCCACAC
